# Kann das Bürgergeld die Lebensmittelkosten einer gesunden Ernährung von Kindern und Jugendlichen decken?

**DOI:** 10.1007/s00103-024-04001-5

**Published:** 2025-01-14

**Authors:** Aziza J. Belgardt, Mathilde Kersting, Kathrin Sinningen, Anjona Schmidt-Choudhury, Frank Jochum, Martin Claßen, Thomas Lücke

**Affiliations:** 1 Forschungsdepartment Kinderernährung (FKE), Universitätsklinik für Kinder- und Jugendmedizin, St. Josef-Hospital, Ruhr-Universität Bochum, Bochum, Deutschland; 2 Abteilung für pädiatrische Gastroenterologie und Hepatologie, Universitätsklinik für Kinder- und Jugendmedizin, St. Josef-Hospital, Ruhr-Universität Bochum, Bochum, Deutschland; 3 Klinik für Kinder- und Jugendmedizin, Ev. Waldkrankenhaus Spandau, Berlin, Deutschland; 4 Deutsche Gesellschaft für Ernährungsmedizin e. V., Berlin, Deutschland; 5 Pädiatrisch-gastroenterologische Praxis, Bremen, Deutschland; 6 Universitätsklinik für Kinder- und Jugendmedizin, St. Josef-Hospital, Ruhr-Universität Bochum, Alexandrinenstraße 5, 44791 Bochum, Deutschland

**Keywords:** Ernährungsbedarf, Kinderernährung, Sozialgesetzgebung, Nahrungsmittelarmut, Optimierte Mischkost, Social law, Food supply, Nutrition, Food poverty, Optimized Mixed Diet

## Abstract

**Einleitung:**

Von Armut betroffene Familien in Deutschland erhalten im Rahmen der Sozialgesetzgebung (SGB) existenzsichernde Leistungen. Das Bürgergeld umfasst auch Regelsätze für Kinder und Jugendliche, gestaffelt in 3 Altersgruppen. Der Bedarf für Ernährung ist die größte Kategorie des Regelsatzes. Die Optimierte Mischkost (OMK) ist ein praxisnahes Konzept einer gesunden Ernährung für Kinder und Jugendliche im Alter von 1–18 Jahren. Herkömmliche Lebensmittel und selbsthergestellte Speisen werden bevorzugt. Ziel des Projektes war es, die Lebensmittelkosten der OMK anhand des zugrunde liegenden 7‑Tage-Speiseplans zu berechnen.

**Methodik:**

Es wurden die niedrigsten Regalpreise der 87 Lebensmittel der OMK im Lebensmitteleinzelhandel (1 Supermarkt, 1 Discounter; November 2022, Bochum) erfasst. Der Ernährungsregelsatz wurde mit den Lebensmittelkosten pro Monat nach den SGB-Altersgruppen sowie nach den Altersgruppen der Deutschen Gesellschaft für Ernährung (DGE) verglichen. Weiterhin wurden die Beiträge der Lebensmittelgruppen zu den Gesamtkosten und der Gesamtenergiezufuhr der OMK aufgeschlüsselt.

**Ergebnisse:**

Die Kosten der OMK wurden mit dem Ernährungsregelsatz des Bürgergeldes für die 3 SGB-Altersgruppen zu 101–109 % gedeckt, für die enger gefassten DGE-Altersgruppen war der Betrag mit wenigen Ausnahmen ebenfalls ausreichend. Hauptsächliche Kostenträger der OMK waren Gemüse/Rohkost, hauptsächliche Energielieferanten waren Brot/Getreideprodukte.

**Diskussion:**

Mit dem Bürgergeld kann der ernährungsphysiologische Bedarf von Kindern und Jugendlichen gedeckt werden, unter der Voraussetzung der Selbstherstellung der Speisen und der Verwendung preiswerter Lebensmittel. Es bleibt zu diskutieren, inwieweit auch mit der Ernährung verbundene Bedürfnisse der sozialen Teilhabe erfüllt werden können.

**Zusatzmaterial online:**

Zusätzliche Informationen sind in der Online-Version dieses Artikels (10.1007/s00103-024-04001-5) enthalten.

## Abstract (en)

**Introduction:**

Families in Germany who live in poverty receive citizens’ benefits (*Bürgergeld*) within the context of welfare legislation. Basic rates for children and adolescents are included in these benefits and staggered into three groups according to age. The need for nutrition is the largest category of the basic rate. The Optimized Mixed Diet (OMD) is a practical concept of a healthy diet for children and adolescents aged 1–18 years. Traditional and home-cooked meals are preferred. The aim of this project was to calculate the food costs of the OMD based on the underlying 7‑day meal plan.

**Methods:**

The lowest shelf prices of 87 different foods from the OMD were recorded during market inspections in different food retailers (1 supermarket, 1 discounter; November 2022, Bochum, Germany). The nutrition basic rate was compared to the total food costs per month in accordance with the age groups of the welfare legislation as well as of the German Nutrition Society (DGE) reference values. Furthermore, the contributions of the food groups were determined based on total costs and total energy intake of the OMD.

**Results:**

The food costs of the OMD are covered 101–109% by the standard nutritional rate of the citizens’ benefits for every age group of the welfare legislation. The costs for the more narrowly defined DGE age groups are covered as well with a few exceptions. The main cost drivers of the OMD were vegetables/raw foods, and the main energy sources were bread/cereal products.

**Discussion:**

The current citizens’ benefits can cover the nutritional requirement of children and adolescents, provided that meals are self-cooked and low-priced foods are used. The question remains to what extent the social needs for participation can be fulfilled as well.

## Einleitung

Viele Familien in Deutschland sind von Armut betroffen. Aktuell gelten ca. 24 % aller unter 18-Jährigen (ca. 2,2 Mio.) als durch Armut oder soziale Ausgrenzung gefährdet [1, 2]. Besonders gefährdet sind Kinder, deren Eltern keinen beruflichen Abschluss besitzen [2]. Einkommensschwache Haushalte erhalten bedarfsabhängige existenzsichernde Leistungen, die in der Sozialgesetzgebung (Sozialgesetzbuch II [SGB II]) geregelt sind. Aktuell lebt jedes fünfte Kind von staatlichen Leistungen zur Existenzsicherung [3].

Im Januar 2023 trat eine Gesetzesänderung in Kraft, mit der das bisherige Arbeitslosengeld II („Hartz IV“) durch das „Bürgergeld“ abgelöst wurde [4, 5]. Im Zuge dessen wurden auch die sogenannten Regelbedarfe für die verschiedenen Haushaltstypen erhöht [4]. Heranwachsende gehören zu den „Familienhaushalten“ und werden in 3 Altersgruppen eingeteilt: Regelbedarfsstufe 4 gilt für Kinder im Alter von 14–17 Jahren, Bedarfsstufe 5 für 6‑ bis 13-Jährige und Bedarfsstufe 6 für 0‑ bis 5‑Jährige [6]. Der Bürgergeld-Regelsatz für Kinder und Jugendliche setzt sich, ebenso wie für Erwachsene, aus verschiedenen Bedarfen zusammen (z. B. Bekleidung und Schuhe, Verkehr). Die größte Bedarfskategorie fasst die Kosten für Nahrungsmittel, Getränke und Tabakwaren zusammen (im Folgenden als „Ernährung“ bezeichnet). Ihr Anteil am Gesamtregelsatz steigt mit zunehmendem Alter von 33 % (0–5 Jahre) auf 44 % (14–17 Jahre) an. Der erstattete Betrag dieser Kategorie müsste die Kosten einer gesunden Kinderernährung decken.

Die Optimierte Mischkost (OMK) ist ein bewährtes Ernährungskonzept für Kinder und Jugendliche, das am Forschungsdepartment Kinderernährung (FKE) entwickelt und evaluiert wurde. Mit der OMK werden die aktuellen wissenschaftlichen Empfehlungen für die Nährstoffzufuhr der Deutschen Gesellschaft für Ernährung (DGE) und der Österreichischen Gesellschaft für Ernährung (ÖGE) in praxisnahe, lebensmittel- und mahlzeitenbasierte Empfehlungen umgesetzt [7, 8]. Zugrunde liegt ein 7‑Tage-Speiseplan, der sich an den Mahlzeitengewohnheiten in Deutschland orientiert [7, 8]. Für die Lebensmittelauswahl gelten 3 einfache Regeln: reichlich Getränke (Trinkwasser) und pflanzliche Lebensmittel, mäßig tierische Lebensmittel, sparsam fett- und zuckerreiche Lebensmittel. Trinkwasser ist Regelgetränk. Herkömmliche, nährstoffreiche Lebensmittel werden bevorzugt. Die Speisen werden in der Regel selbst hergestellt, Fertigprodukte werden vermieden.

Ziel der vorliegenden Arbeit ist es, auf ernährungsmedizinischer Grundlage die Lebensmittelkosten einer gesunden Kinder- und Jugendernährung auf Basis der OMK zu ermitteln und mit dem im Bürgergeld für Kinder und Jugendliche zur Verfügung stehenden Betrag für Ernährung zu vergleichen. Die Ergebnisse können darüber hinaus für die Diskussion möglicher sozialpolitischer Konsequenzen dienen.

## Methodik

### Ermittlung altersbezogener Lebensmittelmengen

Der 7‑Tage-Speiseplan der OMK (Onlinematerial Tab. Z1) bezieht sich auf die Referenzaltersgruppe 4–6 Jahre. Dabei sind die Lebensmittelmengen so kalkuliert, dass die Nährstoffzufuhr (mg [g]/1000 kcal) in allen Altersgruppen von 1–18 Jahren die aktuellen Referenzwerte (DGE; [9]) erreicht, wenn der altersgemäße Energiebedarf gedeckt wird. Die Lebensmittelmengen in den verschiedenen Altersgruppen ergeben sich über Umrechnungsfaktoren basierend auf der altersgemäßen Energiezufuhr (DGE; [7, 8]).

In der Sozialgesetzgebung sind die Altersgruppen bei Kindern und Jugendlichen breiter gefasst als die physiologisch begründeten DGE-Altersgruppen. Ausgehend von der Referenzaltersgruppe (4–6 Jahre) der OMK wurden deshalb Umrechnungsfaktoren für den Energiebedarf der SGB-Altersgruppen ermittelt.

Dazu wurde zunächst jeweils der durchschnittliche Gesamtenergiebedarf (kcal/Tag) für die 3 SGB-Gruppen errechnet. Dieser ergibt sich aus dem Ruhe-Energieumsatz und einem Zuschlag für die körperliche Aktivität (PAL; [10]). Der Bedarf für das Wachstum, der nach dem 1. Lebensjahr mit maximal 1 % angesetzt wird, konnte hier vernachlässigt werden.

Analog zu den DGE-Referenzwerten und den Referenzwerten der Europäischen Behörde für Lebensmittelsicherheit (EFSA) wurde der Ruhe-Energieumsatz unter Berücksichtigung von Körpergröße und Körpergewicht (50. Perzentile nach KiGGS [11]) nach Alter und Geschlecht errechnet [10, 12]. Entsprechend der OMK wurde eine niedrige körperliche Aktivität (PAL 1,4) zur Ermittlung des Leistungsumsatzes gewählt. Dies dient der Prävention von Übergewicht. Schließlich wurde der für Jungen und Mädchen getrennt ermittelte Energiebedarf gemittelt, da das Bürgergeld nicht nach Geschlecht spezifiziert. Mit den Werten für jedes Alter wurden Durchschnittswerte für die Altersgruppen nach SGB errechnet. Analog zum Energiebedarf wurden die notwendigen Lebensmittelmengen des OMK-Speiseplans für die SGB-Altersgruppen berechnet. Der Energiebedarf für das Säuglingsalter konnte mangels Bezugsdaten nicht entsprechend kalkuliert werden [12].

Im SGB werden Säuglinge in die Altersgruppe 0–5 Jahre eingeschlossen. Der Speiseplan der OMK ist nicht für Säuglinge anwendbar, die in den ersten Monaten ausschließlich mit Milch (bevorzugt Muttermilch) ernährt werden und im 2. Lebenshalbjahr zusätzlich Beikost erhalten. Auch rechnerisch kann der Energiebedarf von Säuglingen mangels entsprechender Bezugsdaten nicht nach demselben Verfahren wie bei Kindern und Jugendlichen ermittelt werden. Hier wird deshalb die jüngste Altersgruppe als 1–5 Jahre definiert.

### Ermittlung der Lebensmittelpreise der OMK

Im OMK-Speiseplan werden insgesamt 87 verschiedene Lebensmittel eingesetzt (Onlinematerial Tab. Z2). Für diese Artikel wurde eine „Einkaufsliste“ erstellt, um die Einkaufspreise im Lebensmitteleinzelhandel zu erfassen.

Die Preiserhebung erfolgte im November 2022. Orientiert am Verfahren der Preisermittlung des statistischen Warenkorbes [13] wurden 2 Supermärkte in Bochum einbezogen, ein Discounter und ein Lebensmittelmarkt, die in Zusammenarbeit mit dem Handelsverband Nordrhein-Westfalen Ruhr-Lippe ausgewählt worden waren. Bei einer Begehung wurden jeweils die günstigsten Regalpreise der betreffenden Lebensmittel dokumentiert.

Im OMK-Speiseplan sind die Lebensmittelmengen im verzehrfertigen Zustand angegeben. Die benötigte Einkaufsmenge wurde je nach Lebensmittelgruppe mithilfe von Standardwerten angepasst [14, 15], beispielsweise um nicht verzehrbare Anteile von Obst/Gemüse erhöht oder bei Gewichtszunahme durch Quellen, z. B. Reis, vermindert. Mögliche Lebensmittelabfälle im Alltag mit Kindern konnten hier nicht kalkuliert werden, da diese je nach Haushalt und Alter der Kinder unterschiedlich ausfallen.

### Ermittlung der Ernährungskosten

Zunächst wurden die Kosten der einzelnen Lebensmittel pro Einkaufsstätte für die verzehrten Mengen der verschiedenen Altersgruppen ermittelt. Dies wurde für die SGB-Altersgruppen als auch für die DGE-Altersgruppen durchgeführt. Anschließend wurden durch Addition die Wochenkosten für alle Altersgruppen und für beide Supermärkte erhoben. Zum Vergleich mit den monatlichen Regelsätzen (30 Tage) wurden aus den gemittelten Wochenkosten der Einkaufsstätten die monatlichen Kosten der OMK berechnet. Zuletzt wurden die Beiträge der einzelnen Lebensmittelgruppen der OMK an den Gesamtkosten aufgeschlüsselt und mit den Beiträgen am Gesamtenergiebedarf verglichen.

## Ergebnisse

Tab. 1 zeigt die ermittelten Energiebedarfswerte der SGB-Altersgruppen. Die entsprechenden Umrechnungsfaktoren stellen die Verknüpfung des SGB mit der OMK her und ermöglichen die Ermittlung der Lebensmittelmengen für die Altersgruppen des SGB.

**Tab. 1:** Umrechnung des Energiebedarfs in den Altersgruppen nach Sozialgesetzbuch (SGB) in Relation zur Referenzgruppe „Optimierte Mischkost“ (OMK)

Altersgruppe	Energiebedarf (kcal/Tag)	Umrechnungsfaktor
*Referenzgruppe OMK*		
4–6 Jahre^a^	1350	1
*Altersgruppen SGB*		
1–5 Jahre	1222	0,91
6–13 Jahre	1727	1,28
14–17 Jahre	2381	1,76

^a^ nach Deutsche Gesellschaft für Ernährung (DGE)

**Abb. 1:**
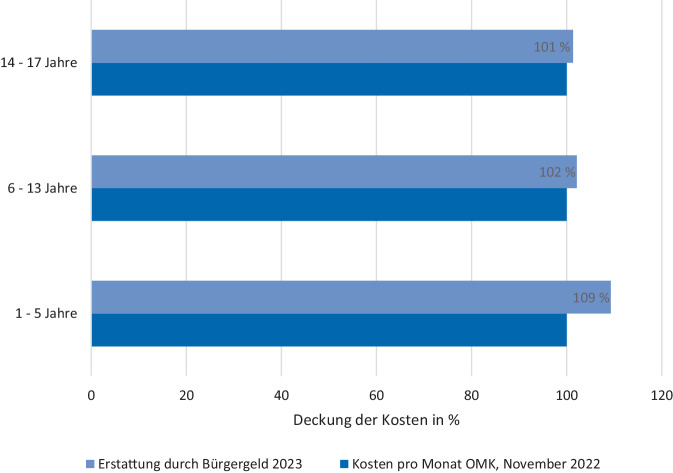
Bedarf für Ernährung im Bürgergeld im Vergleich mit den Kosten der Optimierten Mischkost (OMK). Eigene Abbildung

Tab. 2 zeigt die Ergebnisse der Kostenberechnung der OMK für die Altersgruppen des SGB, spezifiziert für die Einkaufsstätten sowie als Gesamtkosten pro Monat. Mit steigendem Alter verdoppeln sich die Lebensmittelkosten von 96 € (1–5 Jahre) auf 183 €/Monat (14–17 Jahre). In allen Altersgruppen liegt der Monatssatz für Ernährung im Bürgergeld (Stand 2023) knapp über den Lebensmittelkosten der OMK. Die Differenz liegt je nach Altersgruppe zwischen 2,45–8,86 €/Monat bzw. relativ liefert das Bürgergeld zwischen 101–109 % der Kosten der OMK (Abb. 1).

**Tab. 2:** Kosten der Optimierten Mischkost (OMK) im Vergleich zum Bedarf für Ernährung im Bürgergeld

Altersgruppe SGB	OMK-Kosten pro Woche		Durchschnittskosten der OMK pro Monat	Bedarf für Ernährung des Bürgergeldes^a^ pro Monat
	Lebensmittelmarkt	Discounter		
1–5 Jahre	23,96 €	20,59 €	95,49 €	104,35 €
6–13 Jahre	33,53 €	28,76 €	133,50 €	136,37 €
14–17 Jahre	45,96 €	39,37 €	182,87 €	185,32 €

^a^ Stand 2023

**Abb. 2:**
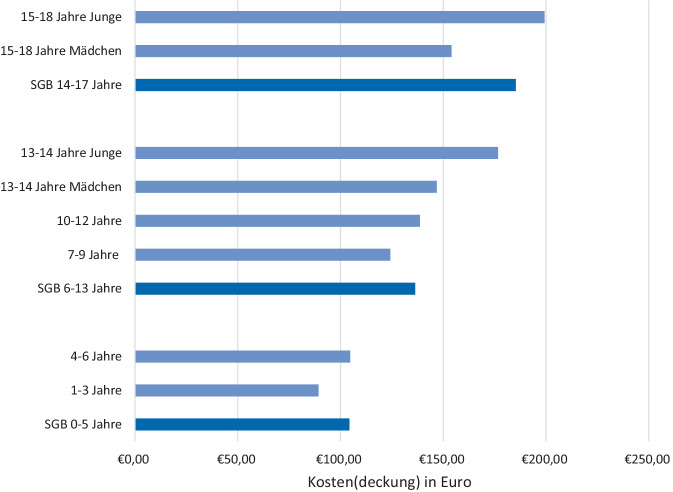
Kosten der Optimierten Mischkost (OMK) nach den Altersgruppen der Deutschen Gesellschaft für Ernährung (DGE) im Vergleich zum Bedarf für Ernährung im Bürgergeld. Eigene Abbildung

Die Regelsätze des Bürgergeldes kommen den errechneten Kosten einer gesunden Kinder- und Jugendernährung in den Altersgruppen der DGE-Referenzwerte sehr nahe (Abb. 2). Ausnahmen sind einige überlappende Jahrgänge und geschlechtsspezifische Unterschiede bei Jugendlichen.

**Abb. 3:**
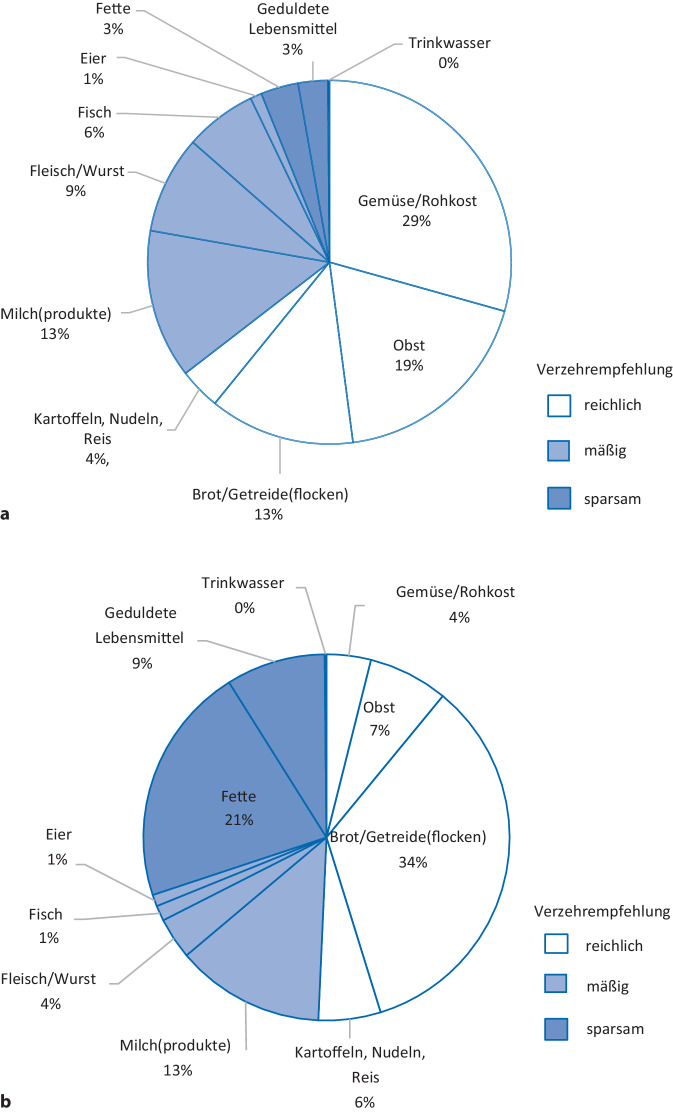
Anteile verschiedener Lebensmittelgruppen **a** an den Gesamtkosten der Optimierten Mischkost (OMK) und **b** an der Gesamtenergiezufuhr der OMK (**b**). Eigene Abbildung. Geduldete Lebensmittel = zucker- und fettreiche Lebensmittel

Der größte Anteil der Kosten der OMK entfällt auf die Lebensmittelgruppen Gemüse/Rohkost und Obst, die zusammen etwa die Hälfte der Gesamtkosten ausmachen (Abb. 3a). Zusammen mit Brot/Getreideflocken und Kartoffeln/Reis/Nudeln entfallen etwa 2 Drittel der Kosten auf pflanzliche Lebensmittel, etwa ein Drittel machen die tierischen Lebensmittel aus. Trinkwasser als Regelgetränk ist kein Kostenfaktor in der OMK. Der größte Anteil an der Energiezufuhr mit der OMK entfällt mit etwa einem Drittel auf die vergleichsweise kostengünstige Lebensmittelgruppe Brot/Getreideflocken (Abb. 3b).

## Diskussion

### Ernährungsmedizinische Aspekte

Eine ausgewogene Ernährung im Kindes- und Jugendalter ist eine wichtige Voraussetzung für Wachstum, Entwicklung, Gesundheit und Leistungsfähigkeit [7]. Sie kann darüber hinaus zur Prävention weitverbreiteter Krankheiten wie Herz-Kreislauf-Krankheiten und Typ-2-Diabetes im Erwachsenenalter beitragen [7, 8]. In der Lebenswirklichkeit besteht ein enger Zusammenhang zwischen dem Ernährungs- und Gesundheitsstatus von Kindern und dem sozioökonomischen Status der Familien [16]: Kinder mit niedrigem Sozialstatus sind häufiger von Übergewicht betroffen als Kinder mit höherem sozialen Status [16, 17]. Auch die Ernährungsgewohnheiten sind ungünstiger, beispielweise wird häufiger Fastfood und Limonade und seltener Obst verzehrt [16–18].

Die hier ermittelten Daten deuten an, dass eine gesundheitsförderliche Ernährung von Kindern und Jugendlichen nach dem Konzept der OMK mit dem Ernährungsregelsatz des Bürgergeldes auch in einkommensschwachen Haushalten grundsätzlich möglich ist, wenn auch knapp.

Auch wenn die Altersgruppen engmaschiger gefasst werden, wie bei den physiologisch begründeten DGE-Altersgruppen, können die Kosten der OMK rechnerisch weitgehend gedeckt werden.

Jugendliche eines bestimmten Alters erscheinen unter- oder überversorgt. Dies könnte mit der breiter gefassten Alterseinteilung der SGB-Altersgruppen erklärt werden. Altersgruppeneinteilungen unterliegen generell einer gewissen Ungenauigkeit und können entwicklungsbedingt große Unterschiede im Energiebedarf von Kind zu Kind nur begrenzt widerspiegeln.

### Lebensmittelauswahl

Der Speiseplan der OMK orientiert sich an einer typischen Kinderernährung in Deutschland, dennoch weicht die Ernährungswirklichkeit vieler Kinder und vor allem Jugendlicher von den Empfehlungen der OMK ab. Weitgehend unabhängig vom sozioökonomischen Status ist z. B. der Verzehr von Gemüse deutlich niedriger, der Verzehr von Fleisch/Wurst und Süßigkeiten höher als in der OMK [17, 19]. In den letzten Jahren ist zwar der Anteil gezuckerter Getränke gesunken [20], aber (Trink)wasser ist noch längst nicht das Regelgetränk in der Kinderernährung.

Bei Orientierung an der OMK könnten beispielsweise höhere Ausgaben für Gemüse durch verminderte Ausgaben für Fleisch teilweise kompensiert werden. Dies wäre auch unter Nachhaltigkeitsaspekten wünschenswert.

Ein Ausschluss von einzelnen, ganzen Lebensmittelgruppen (z. B. Obst/Gemüse) aus der Kinderernährung birgt das Risiko von Nährstoffdefiziten, da sich die Lebensmittelgruppen mit ihren jeweiligen spezifischen Nährstoffprofilen in der Nährstoffversorgung ergänzen.

Besonders im Wachstumsalter muss eine adäquate Energieversorgung gesichert sein. Die bedeutendsten Energielieferanten in der OMK sind Brot/Getreideprodukte (34 %), ihr Anteil an den Kosten ist demgegenüber gering (13 %). In der OMK werden dabei überwiegend nährstoffreiche Vollkornprodukte verwendet. Die vorliegende Aufschlüsselung der Lebensmittelgruppen verdeutlicht insbesondere für Situationen mit knappem familiären Ernährungsbudget, dass das klassische Grundnahrungsmittel Vollkornbrot in der Ernährung von Kindern und Jugendlichen mehr Beachtung verdient.

### Public-Health-Aspekte

Dass Ernährungsempfehlungen nach dem Konzept der OMK weitgehend unabhängig vom sozialen Status umgesetzt werden können, ist aus ernährungsmedizinischer Sicht erfreulich, denn eine gesunde Ernährung ist mit der OMK somit für alle Kinder und Jugendliche zugänglich.

Dieses Ergebnis kann auch die niederschwellige Ernährungsaufklärung erleichtern und das Potenzial der Ernährungsbildung zur Verringerung sozialer Ungleichheit beim Zugang zur gesunden Ernährung von Familien und Kindern ausschöpfen.

Dass die OMK nicht nur gesund, sondern auch kostengünstig ist, ist an bestimmte Kriterien geknüpft, die die Ernährungsqualität und die Nachhaltigkeit der OMK fördern, sozialpolitisch aber zu diskutieren sind. Ein zentrales Kriterium ist der Fokus auf der Bevorzugung herkömmlicher Lebensmittel und der Selbstherstellung der Speisen. Fertigprodukte oder Außerhausessen werden in der Kostenkalkulation des Wochenspeiseplans nicht berücksichtigt; Trinkwasser ist Regelgetränk [7]. Diese Voraussetzungen dürften in der Lebenswirklichkeit der meisten Familien in Deutschland nicht gegeben sein. Allerdings steht Familien in der Grundsicherung ein Zuschuss für die Teilnahme ihrer Kinder an der Gemeinschaftsverpflegung in Kita und Schule zu [21]. Aktuell steht jedoch nur 69 % der Schüler*innen in Deutschland eine Schulverpflegung mit warmen Mahlzeiten zur Verfügung [22]. Die Verpflegung zu Hause ist daher weiterhin von Relevanz.

Besondere kulturelle Ernährungsgewohnheiten konnten hier nicht berücksichtigt werden. Grundsätzlich ist es möglich, spezielle Kostenberechnungen von kulturell adaptierten 7‑Tage-Speiseplänen nach den Regeln der OMK durchzuführen.

Auch unter den Einschränkungen des Bürgergeldes kann Kinderernährung genussvoll bleiben, denn in der OMK wird ein gelegentlicher Verzehr von fett- und zuckerreichen Lebensmitteln, wie Süßigkeiten und Gebäck, die bei Kindern beliebt sind, mit < 10 % der Energiezufuhr „geduldet“, ohne dass die Nährstoffzufuhr darunter leidet oder der Regelsatz überschritten würde.

Wie wichtig soziale Belange für die Bemessung des Ernährungsbetrags im Bürgergeld sind, spricht der Wissenschaftliche Beirat für Agrarpolitik, Ernährung und gesundheitlichen Verbraucherschutz (WABE) an. In seiner Stellungnahme zu Ernährungsarmut unter Pandemiebedingungen betont er, dass mit dem Bürgergeld nicht nur die materielle Ernährungsversorgung gesichert werden muss, sondern auch soziale Armut zu vermeiden ist [23].

Unter sozialen Gesichtspunkten wäre auch der Kostenkalkulationsansatz des vorliegenden Projektes zu diskutieren. Bei der Preiserhebung der Lebensmittel wurden jeweils die niedrigsten Preise der Lebensmittel im Einzelhandel erhoben. Für Familien bedeutet dies einen überdurchschnittlichen Aufwand, da es z. B. erforderlich ist, die Preise der Lebensmittel stets zu vergleichen und Sonderangebote zu bevorzugen. Zudem werden ausschließlich frische Lebensmittel eingeplant und die eigene Zubereitung vorausgesetzt. Dem höheren Aufwand bei der Beschaffung sowie der Zubereitung der Lebensmittel kann jedoch ein möglicher Nutzen gegenübergestellt werden. Eltern und Kinder können durch den vermehrten Umgang mit Grundlebensmitteln sowie der selbstständigen Zubereitung einen alltäglichen Zugang zur gesunden Ernährung bekommen. Die hier durchgeführten Berechnungen beziehen sich auf die Kosten einer aus ernährungsmedizinischer Sicht gesunden Kinderernährung. Bedürfnisse der sozialen Teilhabe müssten zusätzlich betrachtet werden, da Kommensalität (der Akt des gemeinsamen Essens) ein zentrales Bedürfnis des Menschen ist [23, 24].

## Fazit

Die in diesem Projekt erhobenen Daten spiegeln den Status quo der Lebensmittelpreise zum Zeitpunkt November 2022 und der Sozialgesetzgebung beim Bürgergeld Stand Januar 2023 wider.

Auf Basis der Ergebnisse können Konsequenzen für die Sozialgesetzgebung (Ernährungsregelsätze) für die materielle und soziale Ernährungsbedarfssicherung von Kindern und Jugendlichen diskutiert werden, auch unter Beachtung künftiger Änderungen der Lebensmittelpreise (Inflation, Steuern).

Dieses Modell eröffnet darüber hinaus Optionen für modifizierte Kostenszenarien, z. B. mit großzügigeren Einkaufspreisen oder verstärkter Beachtung sozialer Bedürfnisse. Dabei können auch Änderungen der Lebensmittelkosten in den Folgejahren berücksichtigt und mit dem jeweils zur Verfügung stehenden Bürgergeld abgeglichen werden.

## Supplementary Information

Darstellung eines Beispieltages aus der Optimierten Mischkost (OMK); Auszüge aus der Einkaufsliste der Lebensmittel der OMK

## References

[1] Destatis, Statistisches Bundesamt (2023) Gefährdung durch Armut oder soziale Ausgrenzung: AROPE-Indikator nach Geschlecht und Alter im Zeitvergleich. Einkommen und Lebensbedingungen, Armutsgefährung. https://www.destatis.de/DE/Themen/Gesellschaft-Umwelt/Einkommen-Konsum-Lebensbedingungen/Lebensbedingungen-Armutsgefaehrdung/Tabellen/eurostat-armut-sozialeausgrenzung-mz-silc.html. Zugegriffen: 26. Juli 2023

[2] Destatis, Statistisches Bundesamt (2023) Kinder und Jugendliche von Eltern mit niedrigem Bildungsabschluss besonders von Armut bedroht. Pressemitteilung Nr. N045 vom 26. Juli 2023. https://www.destatis.de/DE/Presse/Pressemitteilungen/2023/07/PD23_N045_63.html. Zugegriffen: 27. Juli 2023

[3] ZDFheute (2023) Kindergundsicherung: Das sind die Eckpunkte. https://www.zdf.de/nachrichten/politik/deutschland/kindergrundsicherung-kabinett-100.html?bcrFallback=bcrFallback. Zugegriffen: 18. Dezember 2024

[4] Bundesregierung (2022) Gesetzentwurf. Entwurf eines Zwölften Gesetzes zur Änderung des Zweiten Buches Sozialgesetzbuch und anderer Gesetze – Einführung eines Bürgergeldes (Bürgergeld-Gesetz). Deutscher Bundestag. 20. Wahlperiode. Drucksache 20/3873

[5] Deutscher Bundestag, Online-Dienste (2022) Bundestag stimmt für Bürgergeld-Gesetz. https://www.bundestag.de/dokumente/textarchiv/2022/kw45-de-buergergeld-917430. Zugegriffen: 27. Juni 2023

[6] Sozialgesetzbuch (SGB) Zweites Buch (II) – Bürgergeld, Grundsicherung für Arbeitssuchende – (Artikel 1 des Gesetzes vom 24. Dezember 2003, BGBI. I S. 2954) § 23 Besonderheiten beim Bürgergeld für nicht erwerbsfähige Leistungsberechtigte.

[7] Forschungsdepartment Kinderernährung (FKE) der Universitätskinderklinik für Kinder- und Jugendmedizin Bochum (2019) Empfehlungen für die Ernährung von Kindern und Jugendlichen. Die Optimierte Mischkost. H. Rademann, Lüdinghausen

[8] Kersting M, Kalhoff H, Lücke T (2017) Von Nährstoffen zu Lebensmitteln und Mahlzeiten: Das Konzept der Optimierten Mischkost für Kinder und Jugendliche in Deutschland. Aktuel Ernahrungsmed 42(04):304–315. 10.1055/s-0043-116499

[9] Gesellschaften für Ernährung in Deutschland (DGE),, Österreich (ÖGE), Schweiz (SGE) (2023) DACH-Referenzwerte für die Nährstoffzufuhr, 2. Aufl. Gesellschaften für Ernährung in Deutschland (DGE), Österreich (ÖGE) und der Schweiz (SGE), Bonn

[10] European Food Safety Authority (2017) Scientific opinion: scientific opinion on dietary reference values for energy. EFS2 11(1):3005 10.2903/j.efsa.2013.3005 PMC13159830 42124964

[11] RKI (Robert Koch-Institut) (2011) Beiträge zur Gesundheitsberichterstattung des Bundes. Referenzperzentile für anthropometrische Maßzahlen und Blutdruck aus der Studie zur Gesundheit von Kindern und Jugendlichen in Deutschland (KiGGS), 2. Aufl. Gesundheitsberichterstattung des Bundes, Berlin

[12] Henry CJK (2005) Basal metabolic rate studies in humans: measurement and development of new equations. Public Health Nutr 8(7A):1133–1152. 10.1079/PHN2005801 16277825 10.1079/phn2005801

[13] Destatis, Statistisches Bundesamt, Verbraucherpreisindex (VPI) Was beschreibt der Verbraucherpreisindex. https://www.destatis.de/DE/Themen/Wirtschaft/Preise/Verbraucherpreisindex/Methoden/Erlaeuterungen/verbraucherpreisindex.html?nn=214056. Zugegriffen: 8. Febr. 2024

[14] Souci SW, Fachmann W, Kraut H (2016) Die Zusammensetzung der Lebensmittel. Nährwert-Tabellen, 8. Aufl. MedPharm, Stuttgart

[15] DGH e. V. (2021) Lebensmittelverarbeitung im Haushalt – Teil IV. Hauswirtschaft und Wissenschaft. https://haushalt-wissenschaft.de/wp-content/uploads/2021/06/LMViH_Teil_IV_2021.pdf. Zugegriffen: 8. Nov. 2022

[16] Lampert T, Kuntz B (2019) Auswirkungen von Armut auf den Gesundheitszustand und das Gesundheitsverhalten von Kindern und Jugendlichen. Ergebnisse aus KiGGS Welle 2. Bundesgesundheitsblatt 62:1263–1274. 10.1007/s00103-019-03009-6 10.1007/s00103-019-03009-6 31529186

[17] Brettschneider AK, Barbosa CL, Haftenberger M, Lehmann F, Mensink GB (2021) Adherence to food-based dietary guidelines among adolescents in Germany according to socio-economic status and region: results from Eating Study as a KiGGS Module (EsKiMo) II. Public Health Nutr 24(6):1216–1228. 10.1017/S136898002100001X 33427143 10.1017/S136898002100001X PMC8025090

[18] Moosburger R, Lage Barbosa C, Hafenberger M et al (2020) Fast-Food-Konsum bei 12- bis 17-Jährigen in Deutschland – Ergebnisse aus EsKiMo II. J Health Monit. 10.25646/639435146301

[19] Mensink GBM, Haftenberger M, Barbosa CL () et al (2020) EsKiMo II – Die Ernährungsstudie als KiGGS-Modul. Forschungsbericht. Robert Koch-Institut, Berlin

[20] Mensink GBM, Schienkiewitz A, Rabenberg M, Borrmann A, Richter A, Haftenberger M (2018) Konsum zuckerhaltiger Erfrischungsgetränke bei Kindern und Jugendlichen in Deutschland – Querschnittergebnisse aus KiGGS Welle 2 und Trends. J Health Monit. 10.17886/RKI-GBE-2018-00735586372

[21] Bundesagentur für Arbeit (2024) Leistungen für Bildung und Teilhabe. https://www.arbeitsagentur.de/familie-und-kinder/informationen-zum-bildungspaket. Zugegriffen: 26. Jan. 2024

[22] Nationales Qualitätszentrum für Ernährung in Kita und Schule (2023) Zahlen & Fakten. https://www.nqz.de/schule/zahlen-fakten. Zugegriffen: 27. Aug. 2024

[23] Wissenschaftlicher Beirat für Agrarpolitik, Ernährung und gesundheitlichen Verbraucherschutz beim BMEL (2023) Ernährungsarmut unter Pandemiebedingungen. Stellungnahme. Berlin

[24] Wissenschaftlicher Beirat für Agrarpolitik; Ernährung und gesundheitlichen Verbraucherschutz beim BMEL (2023) Ernährungsarmut in Deutschland. Ein vernachlässigtes Problem, das politisches Handeln erfordert! Ernahr Umsch 5:M304–M307

