# Supplementary material for: Do basic welfare payments in Germany cover the food costs of a healthy diet for children and adolescents?
Source: Bundesgesundheitsblatt Gesundheitsforschung Gesundheitsschutz. 2025 Jan 14;68(2):160–6. [Article in German] doi: 10.1007/s00103-024-04001-5 (PMC11774961; doi:10.1007/s00103-024-04001-5)
Supplement: Supplementary file 1 — Darstellung eines Beispieltages aus der Optimierten Mischkost (OMK); Auszüge aus der Einkaufsliste der Lebensmittel der OMK [file 103_2024_4001_MOESM1_ESM.pdf]

<u>Onlinematerial</u>

Tab. Z1: Beispieltag aus der Optimierten Mischkost (OMK) für die Altersgruppe 4 bis unter 7 Jahre

Frühstück	1. Zwischenmahlzeit	Mittagessen	2. Zwischenmahlzeit	Abendessen
Müsli mit Apfel<br>**150 g** Wasser<br>**100 g** Kuhmilch, 1,5 % Fett<br>**15 g** Mandeln<br>**40 g** Haferflocken<br>**20 g** Cornflakes<br>**7 g** Rosinen<br>**60 g** Apfel	***Wurstbrot mit Weintrauben***<br>**40 g** Vollkornbrot<br>**5 g** Tomatenmark<br>**25 g** Schinkenwurst<br>**50 g** Weintrauben<br>**200 g** Wasser	***Hirsebratlinge mit Kartoffelpüree und Erbsen/Möhren***<br>**15 g** Hirse<br>**5 g** Karotte<br>**5 g** Porree<br>**7 g** Hühnerei<br>**8 g** Schnittkäse, mind. 30 % Fett i.Tr.<br>**3 g** Paniermehl<br>**10 g** Rapsöl<br>**30 g** Erbsen, Konserve<br>**30 g** Karotte, Konserve<br>**120 g** Kartoffeln<br>**5 g** Margarine<br>**20 g** Kuhmilch, 1,5 % Fett<br>**150 g** Wasser	***Obstsalat mit Schokolade***<br>**30 g** Banane<br>**30 g** Ananas<br>**30 g** Erdbeeren<br>**20 g** Schokolade<br>**200 g** Wasser	***Kartoffelsalat***<br>**120 g** Kartoffeln<br>**100 g** Gurke<br>**20 g** Zwiebeln<br>**18 g** Rapsöl<br>**10 g** Essig<br>**200 g** Wasser

Tab. Z2: Auszüge aus der Einkaufsliste - Lebensmittel der Optimierten Mischkost (OMK)

**OMK-Regel**	**Lebensmittelgruppe**	**Lebensmittelbeispiele**
reichlich	Brot/Getreide(-flocken)	Haferflocken
	Brot/Getreide(-flocken)	Cornflakes
	Brot/Getreide(-flocken)	Vollkornbrot
	Brot/Getreide(-flocken)	Weißbrot-Weizenbrot
	Gemüse/Rohkost	Tomaten, Konserve
	Gemüse/Rohkost	Gurke
	Gemüse/Rohkost	Kopfsalat
	Gemüse/Rohkost	Kohlrabi
	Obst	Apfel
	Obst	Erdbeeren
	Obst	Orange
mäßig	Milch(-produkte)	Kuhmilch, 1,5% Fett
	Milch(-produkte)	Schnittkäse, mind. 30% Fett i. Tr.
	Fleisch/Wurst	Schinkenwurst
	Fleisch/Wurst	Rind Hackfleisch
	Fisch	Lachs
sparsam	Öl/Margarine/Butter	Margarine
	Öl/Margarine/Butter	Rapsöl
	Geduldete Lebensmittel	Marmelade
	Geduldete Lebensmittel	Gummibonbons
	Geduldete Lebensmittel	Nuss-Nougat-Creme
